# Long-term effect on adenoid dimensions and craniocervical angulation after maxillary expansion with fixed or functional appliances

**DOI:** 10.4317/jced.58171

**Published:** 2021-06-01

**Authors:** Michele Tepedino, Graziano Montaruli, Francesco Scapato, Michele Laurenziello, Carmela Suriano, Claudio Chimenti, Domenico Ciavarella

**Affiliations:** 1 Department of Biotechnological and Applied Clinical Sciences, University of L’Aquila, L’Aquila, Italy; 2 Department of Clinical and Experimental Medicine, University of Foggia, Foggia, Italy

## Abstract

**Background:**

Maxillary expansion is a common orthodontic procedure that could have a positive effect also on airway patency. The aim of the present study was to evaluate the long-term effects of rapid maxillary expansion (RME) on nasopharyngeal area and cranio-cervical angulation in growing patients, compared to controls treated with a function-generating bite appliance (FGB).

**Material and Methods:**

Sixty patients aged 6-14 consecutively treated with RME or FGB were selected retrospectively and divided into two groups. Lateral cephalograms taken before and after treatment were retrieved, and the nasopharyngeal area, delimited superiorly by a sella-posterior nasal spine (PNS) line and inferiorly by a basion-PNS line, and the cranio-cervical angulation were measured.

**Results:**

The mean observation time was 17.6 ± 8 months. No differences were present between the two groups regarding age and gender. The nasopharyngeal area increased significantly in both groups after treatment, but with no statistically significant difference between them. The cranio-cervical angulation showed no differences within or between groups.

**Conclusions:**

Maxillary deficiency treatment with either RME or FGB was followed by a comparable increase in nasopharyngeal area.

** Key words:**Rapid maxillary expansion, Airway, Nasopharyngeal area, Adenoid.

## Introduction

A reduced transversal width of the maxilla is a common skeletal problem that affects the craniofacial complex, and it is frequently observed in children with abnormal breathing (1). A narrow palate is in fact a characteristic often found in patients with adenoid face (2-4). The routine treatment for transverse maxillary deficiency involves rapid maxillary expansion (RME): a fixed appliance with two or four bands and a jackscrew that applies a displacing force that leads to an opening of the midpalatal suture and outdistances the two maxillary halves (1,5). It has been postulated that opening of the midpalatal suture will result in a lateral displacement of the nasal cavity’s lateral walls, with a consequent increase in nasal airway volume and a reduction in airway resistance (6). For this reason, some authors (7,8) have suggested the use of RME to improve breathing in growing children; however, others have demonstrated the presence of an increase in nasal cavity width after RME (9), which remained stable after 5 years (10). Other authors recognized the change in tongue posture as another possible benefit for airway volume increase after RME (11). In addition, nasal obstructions are followed by changes in head posture that elevates the nose relative to the true vertical (12), and recovers rapidly when decongestants are administered to relieve the obstruction (13). On the other hand, it is not absolutely clear if this increased volume always results in improved breathing: some authors have suggested that this procedure would be helpful when the airway obstruction is located in the anteroinferior part of the nasal cavity, although others also reported beneficial effects in cases where the obstruction was located posterosuperiorly (14). In general, despite some evidence, no strong recommendations can be made for the use of RME with the sole purpose of improving breathing (15).

Most studies that investigated airway patency after RME used volumetric images from cone-beam computed tomographies (CBCTs); however, despite the value of 3D data, some authors have questioned the reliability of such types of measurements, since segmentation procedures, patient positioning and other factors can greatly influence the final outcome (16). Therefore, the value of data retrieved from conventional lateral cephalograms is still relevant for screening purposes, in particular when evaluating the adenoids, which have a simpler morphology, and thus less information is lost on compression in two dimensions (17), especially considering that those exams are routinely needed for orthodontic diagnosis and in light of the As Low As Reasonably Achievable (ALARA) principle (17,18). However, most studies that evaluated the nasopharyngeal space on lateral cephalograms used linear distances from the posterior nasal spine (PNS) to other landmarks (19–22), which do not reflect properly the complexity of the adenoidal and rhinopharyngeal space (17).

The aim of the present study was, therefore, to retrospectively evaluate the change in the adenoidal dimensions and in the cranio-cervical angulation after treatment with RME in growing patients, compared to an active control of matched patients treated with functional appliances. The null hypothesis was that no difference exists in terms of changes in airway space and head position between the two types of treatment for maxillary constriction.

## Material and Methods

This manuscript was prepared according to the STROBE guidelines. The records of all orthodontic patients that were treated from January 2011 to December 2019 at the Orthodontic Clinic, Department of Biotechnological and Applied Clinical Sciences - University of L’Aquila, were screened for the following inclusion criteria:

- Age between 6 and 14 years;

- Diagnosis of a transverse maxillary deficiency;

- Treatment with either an RME or a “function-generating bite” appliance (FGB);

- Lateral cephalograms taken pre- and post-treatment.

Exclusion criteria were allergies in their anamnestic records or previous surgical interventions for airway obstruction. A sample size calculation (G*Power version 3.1.9.2, Franz Faul, Universität Kiel, Germany) revealed that to be able to reject the null hypothesis with a 90% probability and a type I error rate of 0.05, considering a difference between means of 0.25 with a standard deviation of 0.4 (20), a sample size of 55 patients would be needed in each group. Therefore, it was decided that the first 60 subjects that fulfilled the inclusion criteria in chronological order would be included in each group. All procedures were carried out in accordance with the Helsinki Declaration of 1975 and subsequent revisions. All possible attempts were made to contact the patients whose records were selected for inclusion in the study sample, in order to obtain their written informed consent. If this was not achieved after all possible attempts had been made, the need for consent was waived by the Ethics Committee. All the procedures that were followed were approved by the Ethics Committee of the University of L’Aquila (protocol no 42104, ID 13/2020).

Patients treated with an RME appliance were assigned to the study group (RME group), while patients treated with a FGB appliance were assigned to the control group (FGB group). The choice to include an active control group treated with a functional device instead of an inactive control group was due to ethical concerns over delaying orthodontic treatment in a group of growing patients, which would pose a high risk of poorer outcomes.

Patients in the RME group were treated with a two-band bonded Hyrax expander, activated by one turn per day until the desired transversal correction was achieved. After that, the screw was blocked with flowable composite and left in situ for stabilization. Patients in the FGB group were instructed to wear the appliance for 16 hours a day.

**Figure 1:**
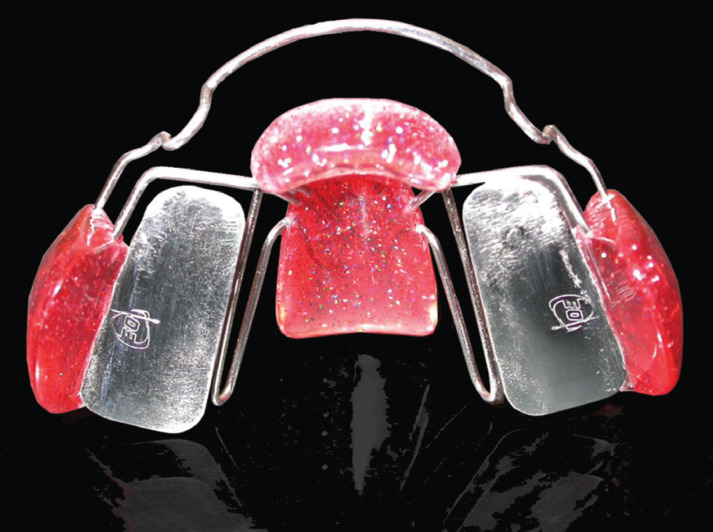
Function generating bite (FGB) appliance, lower occlusal view.

For all included subjects, the pre- (T0) and post-treatment (T1) lateral cephalograms taken in a natural head (Fig. 1) position were retrieved and anonymized with a numerical sequence. The nasopharyngeal area, the A/N ratio and the cranio-cervical angulation were measured on each image.

-Measurement of adenoidal space

**Figure 2:**
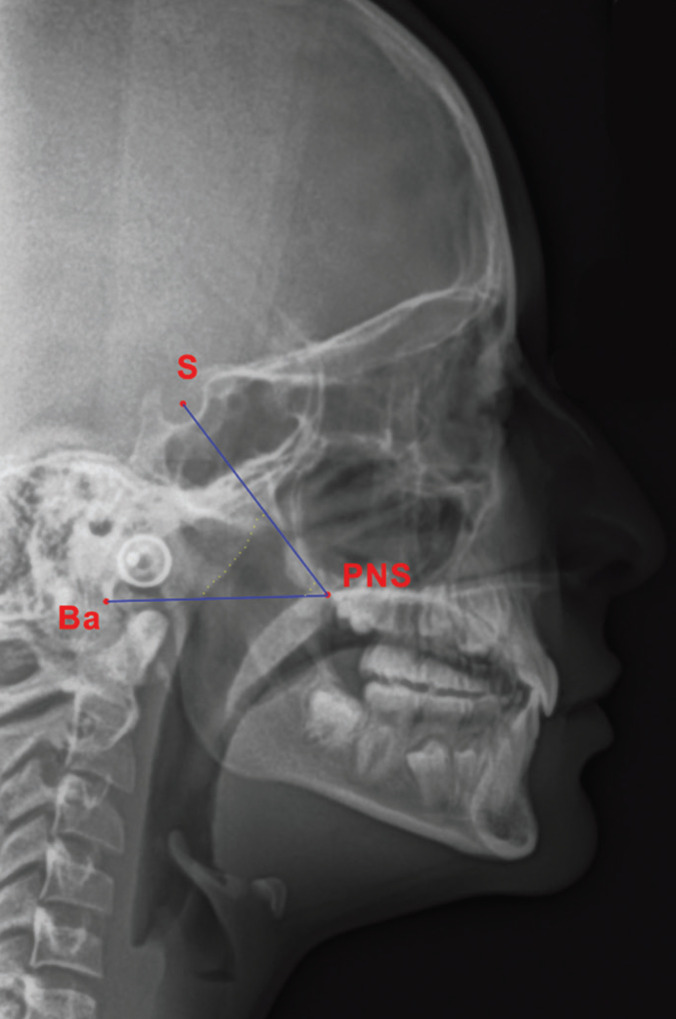
Measurement of nasopharyngeal area. S, sella point; Ba, basion point; PNS, posterior nasal spine point; yellow dotted lines, contour of the soft tissue walls delimiting the nasopharyngeal space.

All the measurements were performed by a single operator who was blinded to the type of treatment the subjects received. The T0 and T1 cephalograms were imported into an image elaboration software program (Adobe Photoshop CS2, Adobe, San Jose, CA, USA) to draw three points: S, the point located at the centre of the sella turcica, PNS, the posterior nasal spine point and Ba, the basion point or the most anterior point of the foramen magnum. Subsequently, two planes were traced on each image: the plane passing through PNS and S, and the plane passing through PNS and Ba (Fig. 2). At this point, the images with the two planes drawn were imported into another software program (ImageJ version 1.5, National Institutes of Health, USA) and calibrated to the actual dimensions using a 30 mm segment positioned on the ruler present in the craniostat of the radiographic appliance. Then, the nasopharyngeal area, delimited by the PNS-S plane, the PNS—Ba plane and the anterior and posterior contour of the upper airways, was measured in mm2 and recorded.

**Figure 3:**
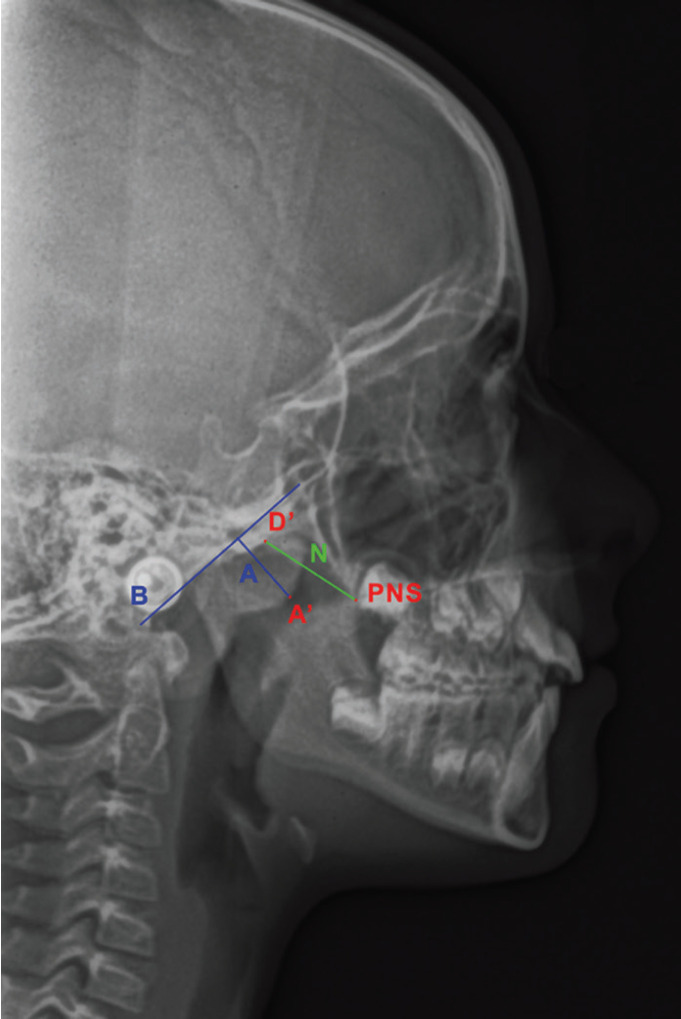
Measurement of the A/N ratio. B, line tangent to the straight part of the basiocciput; A’, the point of maximal convexity of the inferior part of the adenoidal shadow; A, line perpendicular to B and passing through the A’ point; D’, the anteroinferior limit of the sphenobasioccipital synchondrosis, or the intersection between the posteroinferior margin of the pterygoid plates and the inferior margin of the basiocciput; PNS, posterior nasal spine point; N, the line connecting the D’ and the PNS points.

After that, for measurement of the A/N ratio,(23) the images were imported again into the first software program (Adobe Photoshop CS2, Adobe, San Jose, CA, USA) to draw a line (B) tangent to the straight part of the basiocciput and a second line (A) perpendicular to the B line and passing through the A’ point (the point of maximal convexity of the inferior part of the adenoidal shadow. The images with the lines A and B drawn on them were then imported into the ImageJ software program, calibrated to the actual dimensions following the procedure described before, and used to calculate the A/N ratio: the perpendicular distance between the B line and the A’ point, and the distance (N) between PNS and the D’ point (the anteroinferior limit of the sphenobasioccipital synchondrosis, or the intersection between the posteroinferior margin of the pterygoid plates and the inferior margin of the basiocciput) were measured in mm (Fig. 3) and a ratio was calculated dividing the value of A by the value of N.

-Measurements of the cranio-cervical angulation

**Figure 4:**
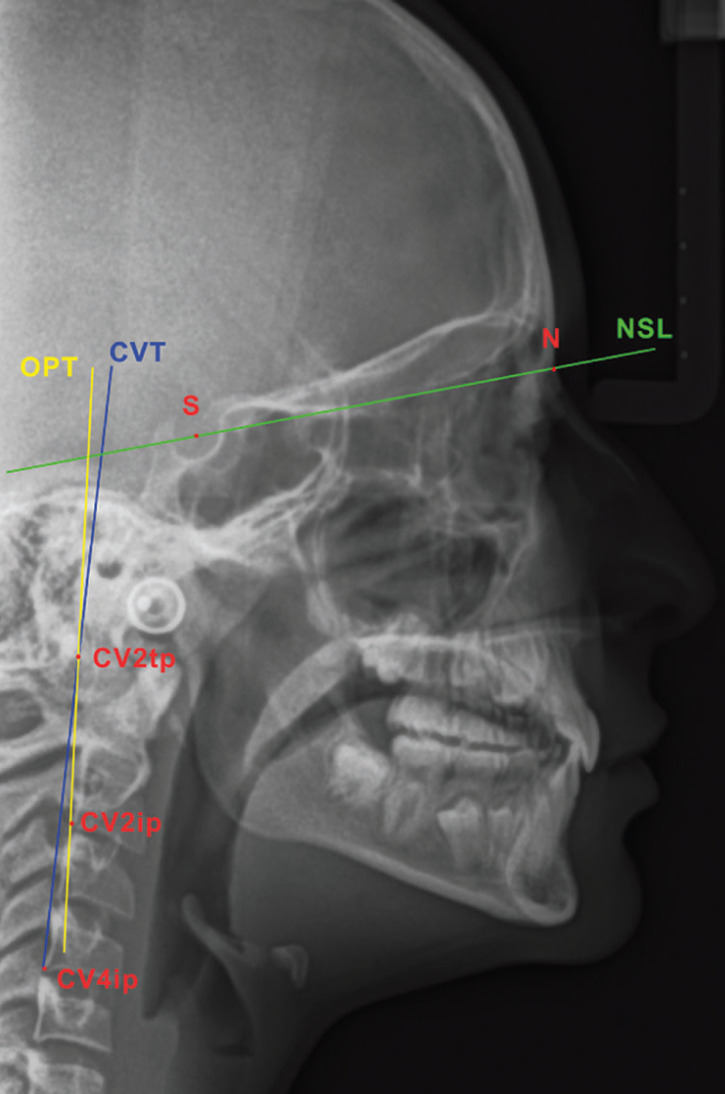
Measurements of the craniocervical angulation. S, sella point; N, nasion point; CV2tp, the most posterior point of the odontoid process; CV2ip, the most inferior and posterior point of the body of the second cervical vertebra; CV4ip, the most inferior and posterior point of the body of the fourth cervical vertebra; NSL, the plane passing through the S point and the nasion point; OPT, the plane tangent to the odontoid process (CV2tp point) passing through the CV2ip point; CVT, the plane tangent to the odontoid process (CV2tp point) passing through the CV4ip point.

The same blinded operator, after importing and calibrating the T0 and T1 images into a measurement software program (ImageJ version 1.5, National Institute of Health, USA) following the already described method, traced three additional planes: NSL, the plane passing through the S point and the nasion point (the most anterior point of the fronto-nasal suture), OPT, the plane tangent to the odontoid process (CV2tp point) passing through the CV2ip point (the most inferior and posterior point of the body of the second cervical vertebra), and CVT, the plane tangent to the odontoid process (CV2tp point) passing through the CV4ip point (the most inferior and posterior point of the body of the fourth cervical vertebra) (24). Subsequently, the angle between the NSL and OPT and the angle between the NSL and CVT were measured in arc degrees and recorded (Fig. 4).

-Error of the method

Twenty-five cephalograms were randomly selected using an online tool, (www.randomizer.org) and the measurements of the nasopharyngeal area and the cranio-cervical angulation were repeated by the same operator at a 30-day interval. The Dahlberg formula was used on the two set of measurements to evaluate the random error, while the presence of systematic errors was evaluated using Bland-Altman plots.

-Statistical analysis

Descriptive statistics for all the variables were calculated. A Shapiro-Wilk normality test was computed to evaluate the data distribution. To evaluate the demographic characteristics of the two samples at baseline, an independent samples t-test or a Mann-Whitney U-test, depending on data distribution, was used to compare the age distribution in the RME and FGB groups at T0, while a Chi-squared test was used to compare the gender distribution between the two groups. To evaluate the within-group T1-T0 difference, an independent samples t-test or a Mann-Whitney U-test, depending on the data distribution, was calculated for each variable. Then, an independent samples t-test or a Mann-Whitney U-test was used for each variable to evaluate the presence of statistically significant differences at T1 between the two groups. The type I error rate was set as 0.05 for each test. The statistical analysis was run using SPSS software (SPSS for Windows v 26, IBM Corp., Armonk, NY, USA).

## Results

The demographic characteristics of the included sample are described in Table 1. There were no differences in age between the two groups (Mann-Whitney U = 1643.5, *p* = 0.958), and the gender distribution across the two groups showed no significant differences (χ = 0.48, *p* = 0.488). The T1 records were taken by mean after 17.6 ± 8 months.

**Table 1:**
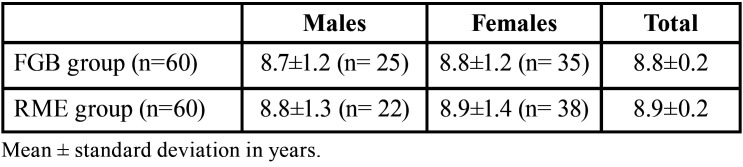
Demographic characteristics of the study sample.

Regarding the error of the method, the random error measured with the Dahlberg formula was 0.88 mm2 for the nasopharyngeal area, 0.18 mm for the measurement of A distance and 0.24 mm for the measurement of N distance, and 0.17° and 0.11° for the measurements of OPT-NSL and CVT-NSL, respectively. The Bland-Altman plots revealed the absence of any systematic errors.

The descriptive statistics for all the variables are reported in Table 2. The nasopharyngeal area increased from T0 to T1, and this change was statistically significant in both groups (Table 3). The A/N ratio decreased in both groups from T0 to T1, with a statistically significant difference (Table 3). On the other hand, the measurements of OPT-NSL angle remained sTable in both groups, while the CVT-NSL angle measurements showed a significant increase in the RME group, but not in the FGB group (Table 3).

**Table 2:**
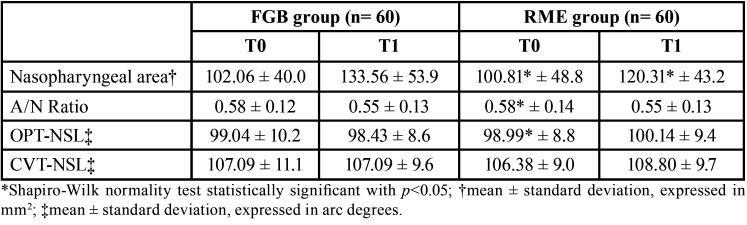
Descriptive statistics for the adenoidal space and the cranio-cervical angulation.

**Table 3:**
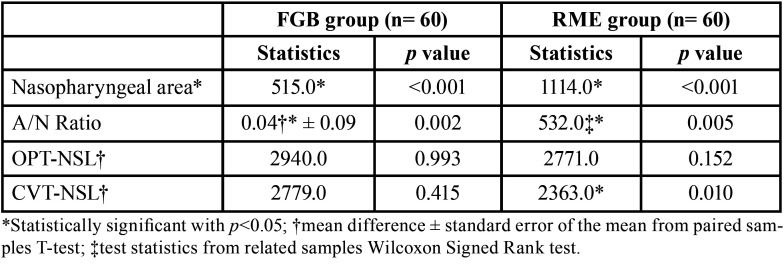
Within groups comparisons (T1-T0) for the adenoidal space and the cranio-cervical angulation.

There were no differences between the two groups regarding nasopharyngeal area, A/N ratio and cranio-cervical angulation at T0 and at T1 (Table 4), therefore the null hypothesis was accepted.

**Table 4:**
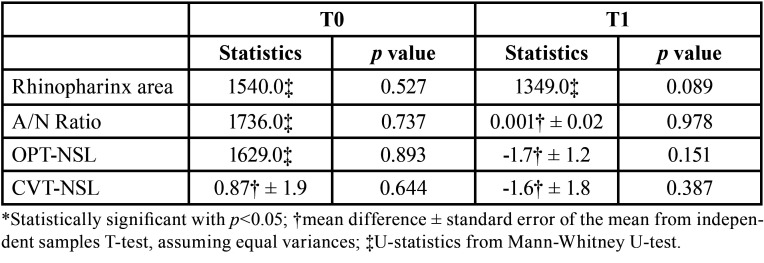
Between groups comparisons for the adenoidal space and the cranio-cervical angulation.

## Discussion

There is still no consensus as to whether the use of CBCTs to calculate airway volume can provide better indications of breathing function than measurements taken on lateral cephalograms (25-29). Measurements made on CBCTs are subject to several confounders, such as the head, body and jaw position at the time of scan acquisition; the respiratory phase and the tongue position. But above all, manual orientation of CBCT images, slice selection, threshold sensitivity and the segmentation protocol are all factors that greatly influence the final measurements and that are seldom considered in the published literature (16,30). For example, inter-examiner differences up to 27% of the measured value were observed for the evaluation of nasopharyngeal volume and minimal cross-sectional area (16). There are many lines of evidence supporting the use of 2D images instead of taking CBCT scans for airway assessment (17,31,32). Sagittal measurements on lateral cephalograms and sagittal slices on CBCT were highly correlated (32) and showed a good correlation with the corresponding evaluated areas on the axial plane (33).

Different types of measurements of adenoidal section and nasopharyngeal space have been proposed in the literature (17), but only one study by Vig *et al*. evaluated the specificity and sensitivity of McNamara’s linear measurement (34) and Schulholf’s area (35) (which is very similar to the area measurement used in the present study, with the only difference being that instead of an S-PNS line, Schulholf used a line perpendicular to the palatal plane passing through the PNS point) in recognizing airway obstruction, reporting a sensitivity of 0.318 and a specificity of 0.833 for McNamara’s linear measurement, and a sensitivity of 0.182 and a specificity of 0.666 for Schulholf’s area (36). However, the low sensitivity shown by Vig *et al*. was probably due to their use of the measurement of nasal resistance as a reference; this way, patients could be considered as having adenoid problems when they actually have rhinosinusitis or simply small nasal cavities. If a patients has no problems with nose breathing, the probability of having hypertrophic adenoids is consequently low, and this will lead to a higher specificity (17). All these results suggest the use of 2D images as a useful guide to the corresponding airway volume for screening and recommending further in-depth otorhinolaryngologic evaluation (31).

According to the results of this study, the nasopharyngeal area increased from T0 to T1 in both groups and the A/N ratio decreased, suggesting the presence of smaller adenoids at T1 (Table 3). Nevertheless, it is possible that this decrease could be due to growth rather than an effect of the two appliances used. Indeed, there is evidence that the coronal and sagittal diameter of the nasopharynx are correlated to age (37), and that the adenoid volume changes with age: their development increases until reaching a peak at 4-5 years of age, then continues until another peak is reached around 9-10 years, following which they start to decrease progressively up to 14-15 years of age (23). The mean age of the present sample at T0 was less than 9 years, and the T1 records were taken a mean of 18 months later; therefore, the likelihood that those patients were in a phase of adenoidal tissue growth peak and that the increase in nasopharyngeal area observed was due to treatment is high. Of course, the only way to prove this statement would be to include a control group of untreated patients; however, it is unethical to postpone a treatment of maxillary expansion in 9-year-old patients by one-and-a-half years with the certainty that the outcome would be worsened because the successful opening of the midpalatal suture is age-dependent (38), and treatment should be carried out ideally with the appliance bonded on deciduous teeth (39).

When the two groups were compared, a comparable increase in nasopharyngeal area and decrease in A/N ratio were observed (Table 4). This result seems to suggest that increasing the diameter of the maxillary arch with either RME or FGB leads to a similar outcome in terms of the adenoidal dimension. While the treatment of RME results in the correction of the transverse occlusal relationship by a high force that acts in a short timespan on both skeletal and dental structures, the FGB promotes a slow expansion using the force of a palatal spring but also the action of the masticatory muscles on the metallic bite planes. Studies have shown that the FGB appliance is able to correct the dental crossbite but also to normalize the masticatory function (40). Therefore, it can be argued that a rapid maxillary expansion and a slow functional expansion can lead to similar outcomes in terms of increasing the nasopharyngeal area. Contradicting results regarding the effects of RME on airways have been found in the literature: while some studies denied a positive effect (19,41), others confirmed an airway increase (15). Anyway, it is difficult to evaluate the clinical significance of such cephalometric changes, even though some authors reported a benefit in terms of the breathing pattern after RME (42).

Concerning cranio-cervical angulation, no differences were found in the OPT-NSL angle before and after treatment and between the two groups (Tables 3 and 4). The CVT-NSL angle showed a significant increase between T0 and T1 only in the RME group, but there were no significant differences when the two groups were compared at both timepoints. This finding is in partial agreement with the results of previous studies, where a more upright head position was observed after RME (15,21,24). A possible explanation could be that since patients with severe respiratory problems were excluded, despite the T1 increase in the nasopharyngeal area, the breathing pattern was not impaired before treatment and did not display noticeable changes.

Regarding the limitations of the present study, the retrospective nature of the protocol should be mentioned, although care was taken to retrieve the samples in a rigid chronological order. The absence of an inactive control group is surely another limitation; however, as mentioned earlier, this methodological choice was dependent on ethical issues.

## Conclusions

Both rapid expansion with RME and slow functional expansion of the maxillary arch with FGB resulted in a post-treatment decrease in adenoidal dimensions and increase in the nasopharyngeal area in growing patients from 6 to 14 years of age, without significant differences between them. The cranio-cervical angulation measured through the CVT-NSL angle increased after treatment only in the RME group but did not display any significant variation between the two groups.
